# Transactions of the Twenty-Sixth Annual Meeting of the Ohio State Medical Society

**Published:** 1872-03

**Authors:** 


*Transactions of the Twenty-Sixth Annual Meeting of the Ohio State Medical Society, held at Cincinnati, April* 4, 5 *and* 6, 1871.

The volume of transactions of this Society comes to us in a very neat and compact form, and contains many papers of interest. The address of the President, Thad. A. Reamy, M. D., of Cincinnati, is an intelligeut and interesting survey of some of the leading questions of the day; the much talked of questions of Medical Education, and Women Doctors come in for a share of treatment, and are handled in a very able manner. Among the many very interesting papers we notice one on "Some points in Uterine Therapeutics," by Edward B. Stevens, M. D., Cincinnati, Ohio.

Dr. J. R. Black, of Newark, O., presents a somewhat lengthy, but very interesting paper on "Sanitary Science" which contains many valuable suggestions. We congratulate the profession of Ohio on the extremely interesting volume of transactions which they have produced.

